# Supplementing the Interference Preservation Hypothesis With Motivational Interviewing–Based Feedback in a Rural Military Veteran With Moral Injury: Formative Case Study

**DOI:** 10.2196/101294

**Published:** 2026-10-22

**Authors:** Justin T McDaniel, Jolie N Haun, Jenna Jamieson, Rebecca Campbell-Montalvo, Dawn Roberts, Lenorris Hughes, Dustin D French

**Affiliations:** 1 School of Human Sciences, Southern Illinois University Carbondale, 475 Clocktower Drive, Carbondale, IL, 62901, United States, 1 6184531865; 2 James A. Haley Veterans' Hospital, Tampa, FL, United States; 3 Division of Epidemiology, Spencer Fox Eccles School of Medicine, University of Utah, Salt Lake City, UT, United States; 4 Department of Emergency Medicine, Morsani College of Medicine, University of South Florida, Tampa, FL, United States; 5 Feinberg School of Medicine, Northwestern University, Chicago, IL, United States

**Keywords:** veterans, college, academic success, moral injury, online education, motivational interviewing

## Abstract

**Background:**

Veterans experiencing moral injury (ie, having witnessed or done something during military service that conflicts with one’s value system) may encounter challenges that affect engagement and learning in online contexts.

**Objective:**

This formative case study examined the feasibility of integrating motivational interviewing (MI)–based delayed feedback, a key tenet of the interference preservation hypothesis, into an online program focused on machine learning instruction for a veteran with self-reported probable moral injury.

**Methods:**

One veteran completed a moral injury assessment and 17 training modules with various checkpoint assessments, followed by a machine learning module incorporating a pretest, MI-based delayed feedback, and a posttest.

**Results:**

The participant completed all instructional and assessment components. Checkpoint scores ranged from 66.7% to 100%, with 7 of 10 checkpoints exhibiting a perfect score. Machine learning performance increased from 90% at pretest to 100% at posttest following MI-based delayed feedback.

**Conclusions:**

These findings demonstrate the feasibility of integrating MI-based delayed feedback within an online data science instruction program and provide formative evidence supporting evaluation in larger samples.

## Introduction

The growing popularity of data science and machine learning has generated new educational opportunities for workforce reskilling, including online learning opportunities for military veterans transitioning to civilian employment. Online microcredential programs offer accessible, modular formats for acquiring such technical competencies [1,2]. However, learners often face educational barriers beyond comprehension of technical concepts, particularly reduced motivation, quantitative learning anxiety, and low self-efficacy, especially with programs that focus on coding [3]. For military veterans, these challenges may be compounded by adjustment stress and the lingering psychological effects of military service [4-6]. Feedback strategies in microcredentials may therefore be important methods for sustaining engagement and improving test performance.

The interference preservation hypothesis (IPH) provides a theoretical basis for the timing of educational feedback [7]. The IPH proposes that delaying corrective feedback may improve learning because immediate feedback can interfere with the learner’s ongoing cognitive processing of newly acquired information. By allowing time to elapse between an initial response and corrective feedback, the learner’s original reasoning and memory may be preserved, creating an opportunity to revisit the problem and engage more actively with the subsequent feedback. Immediate corrective feedback, on the other hand, may prematurely replace or disrupt the cognitive processes involved in generating the learner’s initial response. Thus, delayed feedback may promote retention and transfer by allowing learners to first attempt, process, and reflect on a problem before receiving corrective information. However, the IPH primarily addresses *when* feedback should be delivered rather than *how* that feedback should be communicated. In the present study, we therefore supplemented an IPH-informed delayed feedback approach with motivational interviewing (MI) principles [8] (which will be discussed momentarily) to provide feedback that was not only temporally separated from the learner’s initial attempt but also empathic, reflective, and supportive of autonomy and self-efficacy.

Veterans—the population of focus in this study—exhibit strengths in higher education, such as discipline and determination [9], yet face unique educational barriers during the transition to academic environments. Studies suggest that veterans report higher rates of academic anxiety, concentration difficulties, and lower self-efficacy compared to nonveteran students [10]. These challenges are further associated with psychological factors including mental health problems [11,12]. Veterans may also encounter difficulty adapting from hierarchical, command-based environments to the self-directed, open-ended learning environments typical of online or asynchronous microcredentials [13]. Evidence-based feedback strategies that are supplemented with language centered on autonomy, competence, and self-efficacy may therefore align well with veterans’ motivational needs and learning preferences.

MI is a collaborative, goal-oriented communication method that seeks to strengthen an individual’s motivation and commitment to change [8]. MI has demonstrated moderate effectiveness in promoting behavior change across diverse contexts [14]. The principles of MI (ie, partnership, acceptance, compassion, and evocation) [15] have relevance in education, where autonomy-supportive feedback has been shown to improve engagement and persistence [16]. MI aligns closely with self-determination theory [17], which posits that learning is enhanced when instruction supports autonomy, competence, and relatedness. Therefore, embedding MI-consistent language in assignment feedback may facilitate self-efficacy and intrinsic motivation among veteran learners.

In educational contexts focused on data science, especially when code-based software is used, feedback could be a critical component of learning. However, students new to programming often interpret feedback as evaluative rather than supportive, which leads to reduced persistence and increased anxiety [18]. Effective feedback should therefore balance correction with encouragement and provide structure while maintaining learner autonomy. MI-based feedback, which maintains reflective, affirming, and autonomy-supportive communication, may help learners develop self-efficacy in the face of coding challenges or errors. There is empirical evidence from science, technology, engineering, and mathematics education programs suggesting that autonomy-supportive instructor behaviors are associated with greater task engagement and conceptual understanding [19], implying that MI principles could enhance both motivation and learning outcomes in microcredentials such as the one discussed in this paper.

MI is likely unfamiliar to many technical education audiences, and its application to code-based quantitative training differs in important ways from conventional feedback practices. Traditional feedback in programming and statistics courses is often evaluative, error focused, and directive (ie, typically focusing on correctness, efficiency, or adherence to standards) [20]. Such feedback can inadvertently heighten anxiety or defensiveness, particularly among beginner coders who may interpret corrective statements as judgments of ability. In contrast, MI’s focus on empathy, developing discrepancy, rolling with resistance, and supporting self-efficacy shifts feedback from a corrective approach to a collaborative, autonomy-supportive approach. Instead of simply identifying errors, MI-based feedback acknowledges the learner’s reasoning process, invites reflection on alternative approaches, normalizes difficulty, and reinforces the student’s capacity to improve. These principles from MI align with emerging evidence that affective factors such as confidence, psychological safety, and persistence play a significant role in mastering statistical and computational skills [21]. By integrating MI principles into statistics learning experiences, feedback may serve as not only a tool for correcting code but also a mechanism for strengthening learners’ motivation and resilience on complex technical problems.

The academic challenges experienced by student veterans occur within the broader psychosocial transition from military service to civilian and academic life. The transition theory by Schlossberg [22] provides a useful framework for understanding this process, proposing that individuals’ adaptation to major life transitions is influenced by their situation, personal characteristics, available supports, and coping strategies. For veterans entering higher education, these interacting academic, social, and psychological demands may influence overall well-being and provide important context for understanding mental health problems such as moral injury (which we discuss below) and the potential importance of MI-based feedback.

In the veteran student population, academic challenges are complicated further by mental health problems, as described earlier, which often negatively influence academic performance via their effects on attention, working memory, and emotional regulation [23]. Veterans with more severe mental health symptoms, particularly stemming from moral injury (ie, having witnessed or done something during military service that conflicts with one’s value system) [24-27], report academic challenges [28]. In the classroom, autonomy-supportive and empathetic instructor behaviors are associated with improved persistence and perceived competence among veterans with mental health conditions [29]. MI’s emphasis on nonjudgmental communication and affirmation may mitigate avoidance and defensiveness and, thereby, be an effective intervention for this population.

The present formative case study, similar in nature to a previously published case study [30], aimed to (1) describe the development and implementation of an innovative online instructional approach using MI-based feedback to support a military veteran learning programming and machine learning skills and (2) characterize the participant’s learning trajectory and engagement with the instructional and assessment components. By integrating MI principles, trauma-informed pedagogy, and data science education, this study provides an initial demonstration of the approach and formative evidence to inform its refinement and future evaluation among veteran learners experiencing moral injury.

## Methods

### Sample

The participant in this study (N=1) was recruited from a Midwestern university located in the Illinois Delta Region (a relatively rural region of low socio-economic status) via email. Recruitment messaging invited military veterans to reach out to the principal investigator to express interest in enrolling in a microcredential program focused on machine learning. After the participant self-reported veteran status, prospective participants were provided with an informed consent form to review, sign, and return prior to participation. Moral injury in the study participant was measured and characterized using the Moral Injury Purchase Task (MIPT), which provides a behavioral economic method of screening for moral injury intensity [31].

### Procedures

The microcredential program consisted of 17 sequential modules designed to teach veterans foundational and advanced concepts in data science using the R programming language. The final 3 modules focused on machine learning and were divided into four core components: (1) an overview of machine learning concepts, (2) data preparation and coding practices, (3) model construction and validation, and (4) cross-validation techniques, each representing key competencies necessary for understanding applied machine learning [32].

Following completion of 14 preliminary modules with 10 checkpoints covering data science fundamentals and R syntax (with 10 graded checkpoints), the participant was introduced, step by step, to the process of writing R code for a support vector machine (SVM) [33]. Instructional videos provided an overview of SVM theory, its common use cases, and the underlying logic of classification. Subsequent demonstrations guided learners through practical tasks, including cleaning and recoding datasets, specifying the model equation, and performing both validation and cross-validation procedures.

After watching the instructional video, the participant received a new dataset that paralleled the example used in training accompanied by written directions. The veteran was asked to apply the skills acquired in the tutorial to complete the coding task independently. The assignment required constructing and validating an SVM model. Upon submission, veterans completed an online multiple-choice assessment that served as a pretest. Test items were aligned with the steps presented in the training video, ensuring that the participant could adapt the demonstrated code to a novel scenario.

The pretest consisted of multiple-choice and true-or-false questions assessing both coding accuracy and conceptual understanding, with response options including specific lines of R code and numerical output values (eg, parameter estimates). Automated feedback informed the participant when an answer was incorrect, providing a brief rationale for the error without revealing the correct response. The learner was encouraged to reflect on this feedback, revise their code, and rerun their analyses. After incorporating the feedback, the participant retook the same assessment as a posttest to evaluate learning gains.

### MI-Based Feedback

#### Overview

Test feedback was designed using core principles of MI [8]. The aim of the MI feedback was to provide corrective input while maintaining an empathic, autonomy-supportive tone consistent with trauma-informed pedagogy. Rather than simply indicating whether responses were correct or incorrect, each feedback message was scripted to evoke reflection, encourage problem-solving, and reduce defensiveness. Four specific MI principles were applied during feedback design (Table 1).

**Table 1.** Theoretical basis for motivational interviewing (MI)–style feedback in an online technical training context.

	Principle 1: express empathy	Principle 2: develop discrepancy	Principle 3: roll with resistance	Principle 4: support self-efficacy
Description	Acknowledge and validate the learner’s reasoning	Help learners recognize the gap between their current approach and the desired outcome	Respond to frustration with compassion and non-judgment	Reinforce confidence and persistence
Example feedback	“It’s easy to trace your train of thought here. The training video included the exact function that you selected, but it’s not appropriate in this context.”	“To achieve the correct solution, you should consider looking at the file extension on the practice dataset. What type of file extension is used for the dataset? Understanding that will enable you to import the dataset in R correctly.”	“Solving this complex task is difficult and requires some practice.”	“However, through practice, you will be able to master this task!”
Relevance to this study	This principle centers on MI’s emphasis on empathic communication that promotes psychological safety and engagement	This principle encourages self-discovery and active reasoning, which aligns with MI’s goal of promoting intrinsic motivation via awareness of discrepancy	This principle centers on MI’s aim to de-escalate resistance via understanding instead of correction, which prevents defensive reactions and engagement	This principle affirms knowledge and ability, and encourages continued effort, which brings about self-efficacy

#### Principle 1: Expressing Empathy

Feedback statements were structured to acknowledge and validate the learner’s reasoning process before providing correction. For example, responses such as “It’s easy to trace your train of thought here. The training video included the exact function that you selected, but it’s not appropriate in this context” were used to demonstrate understanding of the participant’s logic while gently highlighting the error. This feedback aligned with MI’s emphasis on empathic communication to promote psychological safety and engagement.

#### Principle 2: Developing Discrepancy

Learners were guided to recognize the gap between their current coding approach and the strategy required to achieve an accurate outcome. Illustrative feedback included prompts such as “To achieve the correct solution, you should consider looking at the file extension on the practice dataset. What type of file extension is used for the dataset? Understanding that will enable you to import the dataset in R correctly.” This method encouraged self-discovery and active reasoning, which is consistent with MI’s goal of eliciting intrinsic motivation through discrepancy awareness.

#### Principle 3: Rolling With Resistance

In situations in which repeated errors or frustration were evident, feedback was written to avoid confrontation or evaluative language. Instead, responses conveyed compassion and normalized difficulty as follows: “Solving this complex task is difficult and requires some practice.” The tone of the feedback was designed to prevent defensive reactions and sustain engagement, which reflects MI’s strategy of de-escalating resistance through understanding rather than correction.

#### Principle 4: Supporting Self-Efficacy

Finally, each feedback sequence concluded with statements designed to reinforce confidence and persistence. For example, the following statement was included in one of the test items: “However, through practice, you will be able to master this task!” This piece of feedback served to affirm competence and encourage sustained effort, as well as enhancing learning self-efficacy.

### Measures

The SVM module assessment consisted of a 10-item pretest and posttest designed to evaluate participants’ applied understanding of key R programming and machine learning procedures. Items assessed ten domains of skill acquisition: (1) importing datasets, (2) managing missing data, (3) creating composite indexes and calculating their means, (4) recoding outcome variables into categorical forms using evidence-based thresholds, (5) subsetting datasets to select specific variables or columns, (6) generating visualizations, (7) partitioning data into training and testing sets, (8) estimating training models, (9) conducting parameter tuning and cross-validation, and (10) reporting model accuracy.

Each item required the selection of the correct R function, syntax, or output value. For example, one question asked the following: “How many observations should be included in the training dataset if we randomly select 60% of observations from the dataset?” MI-based feedback for this particular question is provided in Figure 1. Responses to each question in the 10-item exam were scored dichotomously (1=correct and 0=incorrect), yielding a total possible score between 0 and 10.

**Figure 1.**
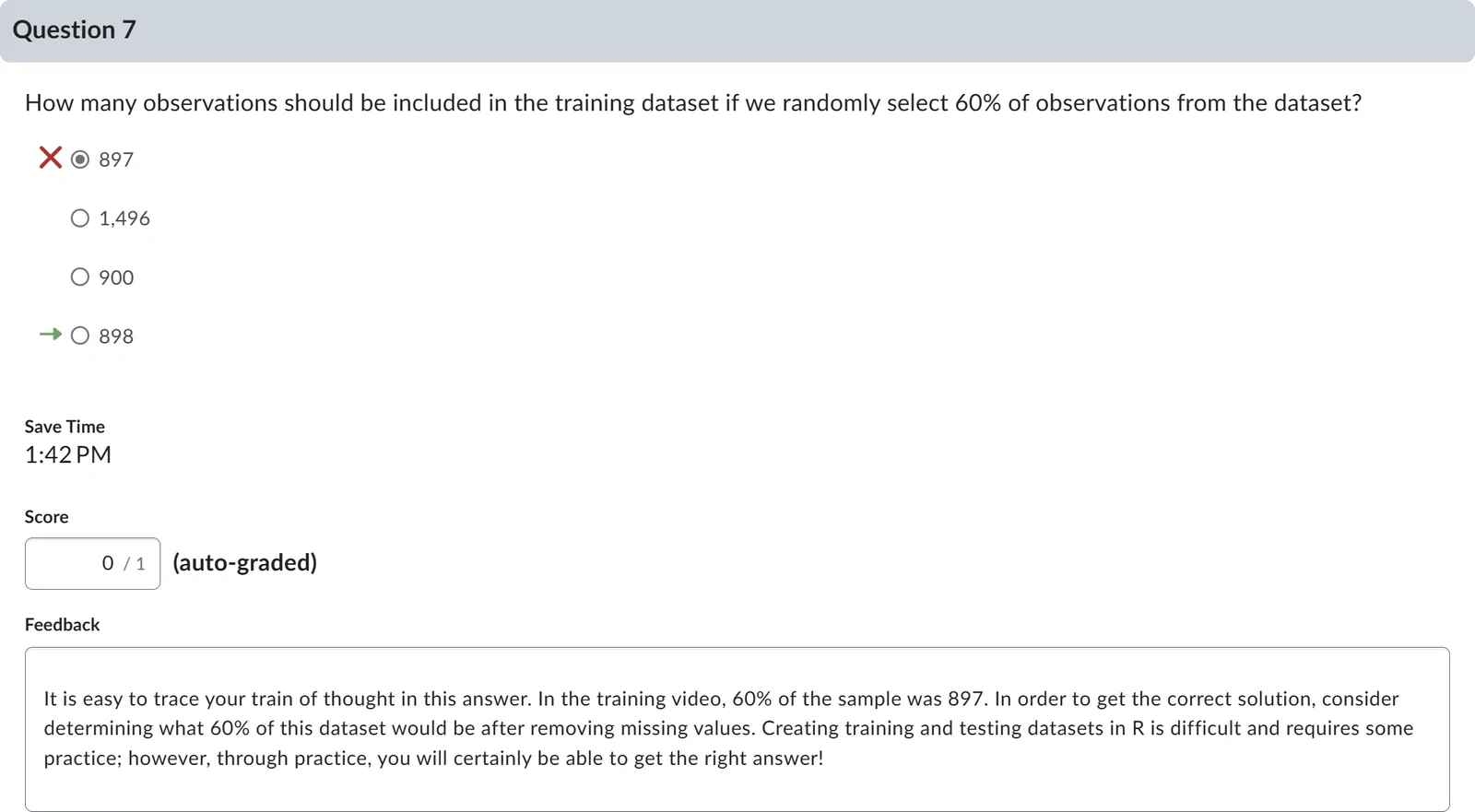
Example test question with motivational interviewing–style feedback included on an incorrect answer within the online machine learning module.

### Statistical Analysis

The participant’s moral injury status was characterized by estimating a modified exponential demand curve to responses on the MIPT, which is documented in Equation 1 [34]:


(1) $$Q=Q_{0}\times 10^{\left\{k\left(e^{\left\{-\alpha Q_{0}C\right\}}-1\right)\right\}}$$

Previous studies have shown that moral injury may be clinically significant when moral injury intensity (ie, the response value on the first question of the survey) is greater than or equal to 30 [31].

Performance across the 10 training checkpoints was summarized using individual checkpoint scores (ie, reported as a percentage of correct responses) and visualized longitudinally to characterize the participant’s learning trajectory before completion of the final machine learning module. Performance on the machine learning pretest and posttest was reported descriptively as the percentage of correct responses, and within-participant change was calculated as the absolute percentage-point difference between pretest and posttest scores.

### Ethical Considerations

The Institutional Review Board of Southern Illinois University approved the protocol for this study (22043). The participant provided written consent to enroll in the study.

## Results

The participant completed all questions of the MIPT, permitting examination of whether the task generated an interpretable individual-level characterization of moral injury status. Hypothetical consumption of services to heal moral injury remained at the maximum response of 60 units across the first 8 price levels, providing evidence of severe and possibly clinically significant (ie, intensity ≥30) moral injury (Figure 2). As price increased, consumption subsequently decreased to 30, 15, 5, and 3 units. Consumption reached 0 at US $10,240 and remained at 0 across all higher prices, identifying US $10,240 as the participant’s observed break point.

**Figure 2.**
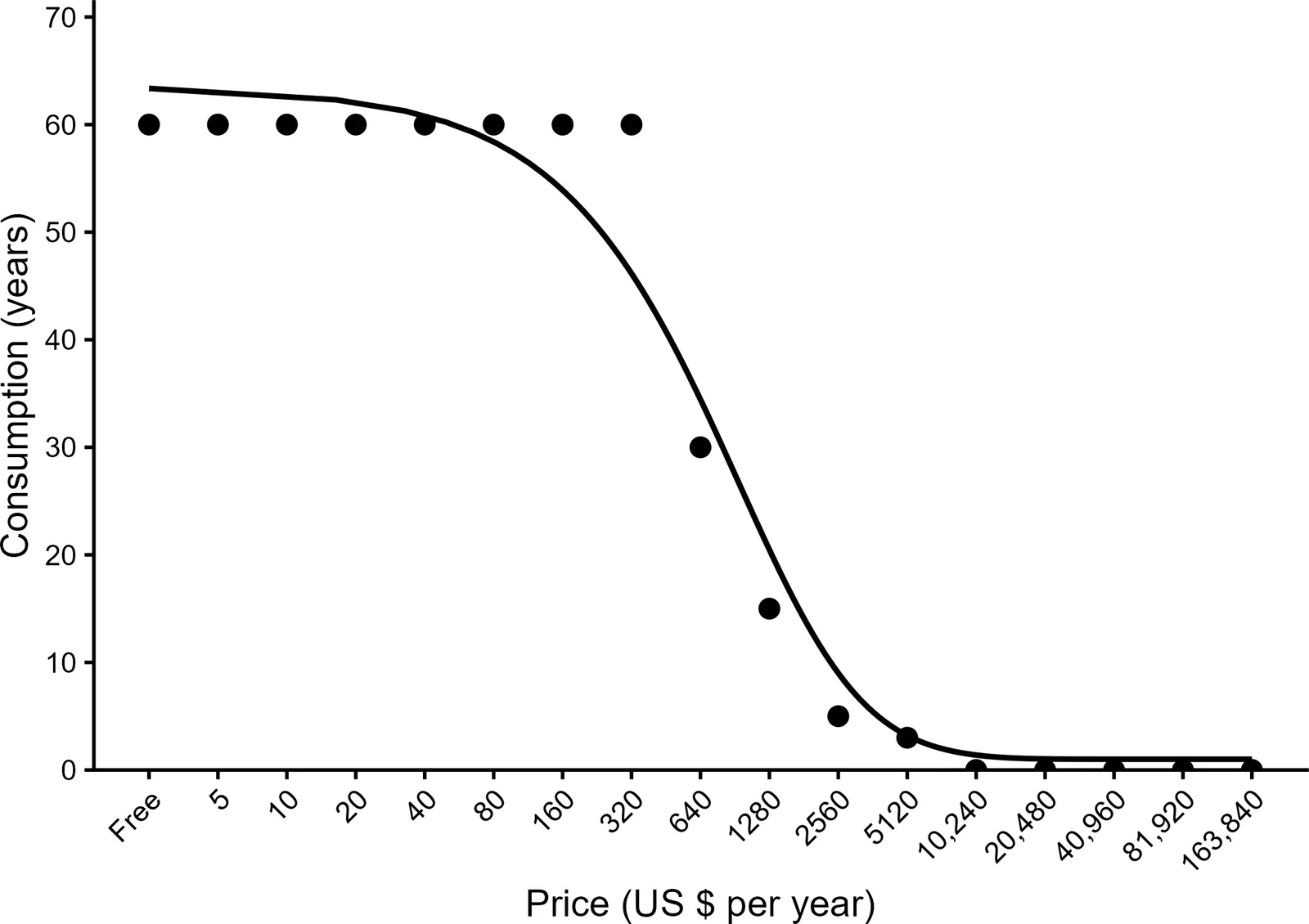
Characterization of moral injury in the case study participant.

The participant completed all training modules and the 10 associated checkpoint assessments before progressing to the final machine learning module. Performance across the checkpoints was generally high, but prior to the infusion of MI-based feedback, the participant received a 67% (below a C– grade) on 3 checkpoints. Following checkpoint 5, performance remained at 100% across checkpoints 6 to 10 (Figure 3). Ignoring course performance, the participant demonstrated successful progression and engagement through the training sequence.

**Figure 3.**
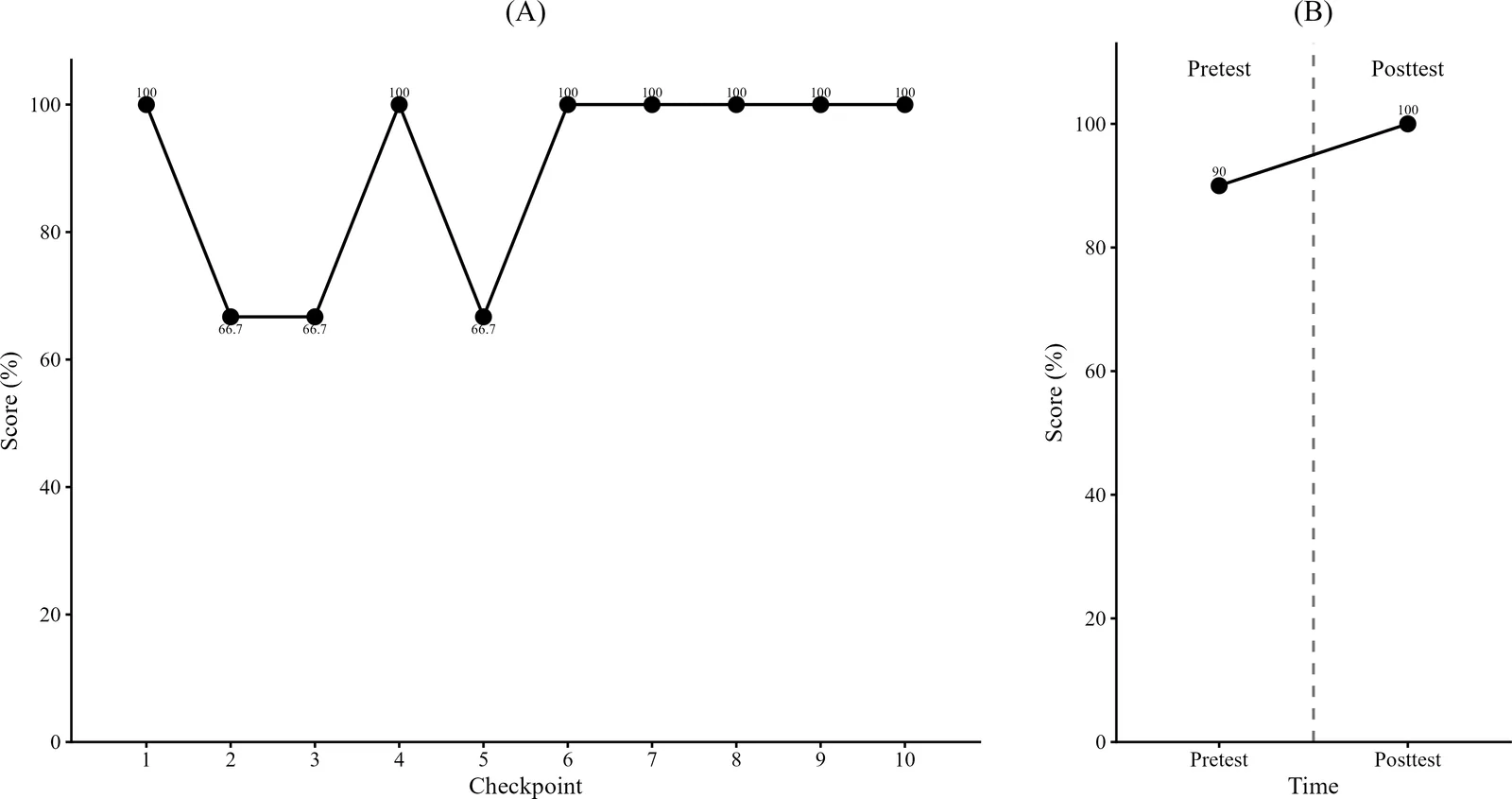
Single participant engagement and learning trajectory through the online training modules (A), including scores on the final module posttest following provision of motivational interviewing–based feedback on the pretest (B).

The participant subsequently completed the final machine learning module. The participant scored 90% on the initial pretest. Following exposure to the individualized MI-based delayed feedback, the participant scored 100% on the posttest, corresponding to an absolute increase of 10 percentage points. Thus, the feedback approach was successfully delivered within the learning management system and was followed by score increases at the posttest.

## Discussion

Overall, findings from this formative case study demonstrate the feasibility of implementing MI-based delayed feedback (ie, a supplement to the IPH) within an online training program for a veteran with self-reported severe moral injury. The participant successfully completed the training sequence with some variability in checkpoint performance (ie, A+ on 7 checkpoints and D on 3 checkpoints), received the MI-based feedback as designed, and demonstrated a 10 percentage-point increase in course performance from pretest (90%) to posttest (100%). This within-participant change provides preliminary evidence that the feedback approach can be integrated into the learning experience while maintaining participant engagement and supporting successful module completion. Given the formative N=1 design, the observed change cannot establish intervention efficacy or a causal effect of MI-based feedback. Rather, these findings provide an initial demonstration of the intervention and assessment procedures and support further evaluation in a larger sample.

Consistent with findings from Lei et al [16], who reported that MI-based interventions improved learners’ motivation and performance across educational settings, the present results suggest that embedding MI principles within test feedback may help veterans learn how to code in R. The observed improvement in posttest scores in this study corresponds with prior evidence that empathic, nonjudgmental communication with individuals may enhance persistence and reduce defensiveness [8].

It is possible that incorporating MI principles into instructional feedback holds promise as a pedagogical approach for supporting learning in statistics and data science education. Although firm conclusions cannot be drawn from this formative case study, MI-based feedback, which is characterized by empathy, guided discrepancy, and autonomy-supportive dialogue, may help foster cognitive engagement and reduce affective barriers for some learners. In programming and statistics learning contexts, such an approach has the potential to support learners’ persistence, self-regulation, and confidence when working through complex analytical tasks, but additional controlled research is needed to evaluate these possibilities. The present findings tentatively align with emerging work on affective-cognitive feedback models in statistical computing, particularly for adult and veteran learners who may vary widely in motivational and psychological characteristics. More broadly, these results contribute preliminary data to the growing literature on trauma-informed and autonomy-supportive pedagogies [35], which indicates that MI-informed feedback warrants further investigation as a potential strategy for enhancing learning experiences in quantitative coursework.

Several limitations should be considered when interpreting the findings of this study. First, the small sample size associated with this case study limits generalizability. Second, the participant was recruited from a single Midwestern university, which may not reflect the broader population of veterans or learners in other educational settings. Third, moral injury symptoms were assessed via self-report screening rather than clinical diagnosis. Future research using larger, more diverse samples and randomized designs is warranted to validate and extend these findings across broader statistical and computational learning contexts.

Ultimately, the present formative case study provides an initial demonstration of how feedback grounded in empathy, guided reflection, and support for self-efficacy can be operationalized within a quantitative, code-intensive learning environment framed around the IPH. These formative findings support further refinement and evaluation of MI-informed pedagogy as a potentially useful approach for supporting veteran learners experiencing moral injury.

## Abbreviations

IPH: interference preservation hypothesis
MI: motivational interviewing
MIPT: Moral Injury Purchase Task
SVM: support vector machine
